# Effector T helper cell populations are elevated in the bone marrow of rheumatoid arthritis patients and correlate with disease severity

**DOI:** 10.1038/s41598-017-05014-8

**Published:** 2017-07-06

**Authors:** Shufeng Li, Han Yin, Kaining Zhang, Ting Wang, Yun Yang, Xinguang Liu, Xiaotian Chang, Ming Zhang, Xinfeng Yan, Yanjun Ren, Wenping Pan, Lei Zhang

**Affiliations:** 1 Department of Orthopedics, Shandong Provincial Qianfoshan Hospital, Shandong University, Jinan, 250014 P.R. China; 2 Department of Orthopedics, Liaocheng People’s Hospital and Clinical School of Taishan Medical University, Liaocheng, 252000 P.R. China; 3 Department of Obstetrics and Gynecology, Shandong Provincial Qianfoshan Hospital, Shandong University, Jinan, 250014 P.R. China; 4 Department of Hematology, Qilu Hospital, Shandong University, Jinan, 250012 P.R. China; 5 Medical Research Center, Shandong Provincial Qianfoshan Hospital, Shandong University, Jinan, 250014 P.R. China; 6 Department of Rheumatology, Shandong Provincial Qianfoshan Hospital, Shandong University, Jinan, 250014 P.R. China

## Abstract

This study is to investigate the frequencies of T-helper (Th)22, Th17 and Th1 cells and the levels of related cytokines in subchondral bone marrow in patients with rheumatoid arthritis (RA). Bone marrow and peripheral blood samples were collected from RA, osteoarthritis (OA) patients and healthy controls. The frequencies of Th22, Th17, and Th1 cells were examined by flow cytometry. Levels of interleukin (IL)-22, IL-17 and IFN-γ were examined by ELISA. Disease Activity Score in 28 joints (DAS28) of RA patients were analyzed. Bone marrow Th22, Th17 and Th1 cells in RA patients were markedly increased comparing to OA or healthy controls. Each T cell subset in bone marrow was elevated comparing with that in the peripheral blood in RA patients. Consistently, plasma levels of IL-22 and IL-17 were elevated in RA patients, and the elevation was more notable in the bone marrow than in the peripheral blood. Additionally, the percentages of Th22, Th17 and Th1 cells as well as the levels of IL-22 and IL-17 in bone marrow were positively correlated with DAS28. These results suggest that local pro-inflammatory Th cells are elevated in bone marrow, which may play an important role *in situ* in RA and contribute to the pathogenesis of in RA.

## Introduction

Rheumatoid arthritis (RA) is a chronic inflammatory autoimmune disease characterized by destruction of articular cartilage and bone destruction. The interactions between immune cells and bone cells contribute to pathogenesis of RA^1, 2^. Activated CD4+ T cells have been implicated in bone damage associated with chronic inflammation^3^. In autoimmune arthritis, the generation of osteoclasts is directly and indirectly regulated by CD4+ T cells that migrate to bone lesion and contribute to bone destruction^4, 5^. Th17 cells are important inflammatory CD4^+^ T cells that secrete interleukin (IL)-17A^6^. Th17 cells are shown to be increased in the peripheral blood and synovial fluid of RA patients, suggesting the pathogenic role of Th17 in RA^7–10^. In addition, Th17 cells act as osteoclastogenic helper T cells^11^. IL-17, the main effective cytokine of Th17 cells, is associated with increased osteoclastogenesis by inducing receptor activator of nuclear factor-κB ligand (RANKL) expression on osteoblasts in RA^3^. Th22 cells are the subset of inflammatory CD4+ T cells, which are characterized by the production of IL-22, but not IL-17 or IFN-γ^12, 13^. IL-22, a main signature cytokine of Th22 subset, promotes osteoclastogenesis and enhances bone destruction in arthritic mice^14, 15^. Elevated serum IL-22 is associated with disease activity in RA patients^16^, and disease severity is shown to be markedly reduced in IL-22^−/−^ mice with collagen-induced arthritis^17^. Recently, roles of IL-22 are highlighted in pathogenesis and therapy of RA^18^. Furthermore, serum levels of IL-22 are related to the radiographic progression of RA patients^19^, suggesting a pathogenic role of IL-22 in bone destruction of RA patients. Neutralization of IL-22 results in reduced number of inflammatory cells and has similar effect on bone erosion^20^. Tumor necrosis factor (TNF)-α, another crucial effective cytokine of Th22 cells, is a main pathogenic cytokine in RA. TNF-α has destructive effect on bone^21^. In addition, TNF-α produced by aberrant T helper cells is involved in the pathogenesis of bone loss in RA^22^.

Before the discovery of Th17 and Th22 subsets, researches on inflammatory CD4+ T cells in RA are focused on Th1 cells, which secrete IFN-γ as their main effector cytokine. RA is considered a Th1-associated disease^23^, and abundant Th1 cells are observed in synovial fluid of RA patients^24^. Activated Th1 cells intensify osteoclastogenesis despite of the anti-osteoclastogenic effect of IFN-γ. It is well known that systemic inflammation results in increased circulating inflammatory immune cells. The profiles of Th22, Th17 and Th1 cells in peripheral blood of RA patients have already been analyzed in our previous studies^25, 26^. Local bone erosion is generally driven by inflammatory synovium in RA. In the past, most studies on RA are concentrated on T helper cell subset in peripheral blood, synovial fluid and synovium. Recent attention has been focused on the subchondral bone of the joints. According to magnetic resonance imaging (MRI) of RA joints, bone marrow is under attack and associated with bone erosion in the early course of disease, when synovitis does not spread to subchondral bone tissues across the relatively intact cartilage^27, 28^. Therefore, we speculate that pathologic changes of bone marrow in joint destruction are independent to a certain extent, and bone marrow may play a certain role in the pathogenesis of RA. Relatively little is known about the profiles of CD4+ cell subset in subchondral bone marrow in RA. The profiles of T helper subset in peripheral blood cannot exactly reflect the local bone condition of RA. In order to investigate immune changes *in situ* and to understand the pathogenic mechanism, we detected the frequencies of Th1, Th17 and Th22 cells in bone marrow of RA patients and analyzed their correlation with RA activity.

## Materials and Methods

### Patients

A total of 40 patients who were diagnosed with active RA according to the criteria of the American College of Rheumatology were included in the present study^29^. Active RA was defined by Disease Activity Score in 28 joints (DAS28) ≥2.6^30^. The patients consisted of 33 women and 7 men, with mean disease duration of 12.8 ± 6.5 years. The mean age of the patients was 62.2 ± 7.0 years (Table 1). Nine osteoarthritis (OA) patients (7 females and 2 males; mean age, 63.8 ± 3.8 years) were recruited as disease controls. In addition, 9 trauma patients (7 females and 2 males; mean age, 62.9 ± 4.7 years) who had no systemic inflammatory disease or immune abnormalities were included as healthy controls. Paired samples of bone marrow and peripheral blood were obtained from the same RA patient, OA patient and healthy subject. Bone marrow samples were obtained from RA and OA patients during total knee arthroplasty. None of OA patients and healthy controls had any systemic inflammatory diseases. Enrollment took place between March 2013 and December 2015 in the Department of Orthopedics, Shandong Provincial Qianfoshan Hospital, Shandong University, China. Our research has been approved by the Medical Ethical Committee of Shandong Provincial Qianfoshan Hospital, Shandong University. Informed consent was obtained from each patient before being included in the study. All experiments were performed in accordance with relevant guidelines and regulations.

**Table 1:** Demographic and clinical characteristics of rheumatoid arthritis patients

Characteristics	Values
No. of patients	40
Age (year)	62.2 ± 7.0
Sex (male/female)	7/33
Disease duration (year)	12.8 ± 6.5
RF positive	31/40 (77.5%)
Anti-CCP positive	28/40 (72.5%)
ESR (mm/h)	72.9 ± 27.8
CRP (mg/L)	56.7 ± 34.2
No. of swollen joints	12.2 ± 6.3
No. of tender joints	13.7 ± 7.2
DAS28	6.8 ± 1.3

Note: RF, rheumatoid factor; anti-CCP, anti-cyclic citrullinated peptide antibody; ESR, erythrocyte sedimentation rate; CRP, C-reactive protein; DAS28, Disease Activity Score in 28 joints.

### Sample preparation

Bone marrow blood (5 ml) was aspirated from the tibial proximal epiphysis by needle puncture at the time of operation. Simultaneously, 5 ml venous blood was collected from the same patient. During bone marrow blood collection, peripheral blood contamination was considered to be probable. Therefore, we performed a preliminary experiment by examining T cell subsets in different volumes of bone marrow blood in the same patients. When the volume of bone marrow blood did not exceed 5 ml, the frequencies of T cell subsets showed no significant difference and the bone marrow blood was not considered to be contaminated by peripheral blood (data not shown).

### Flow cytometry

Levels of intracellular cytokines were studied by flow cytometry. Briefly, heparinized whole blood (400 μl) was mixed with an equal volume of Roswell Park Memorial Institute 1640 medium and incubated for 4 h at 37 °C under 5% CO_2_ in the presence of 25 ng/ml phorbol myristate acetate (PMA), 1 μg/ml ionomycin, and 1.7 μg/ml Golgiplug (Monensin; all from Alexis Biochemicals, San Diego, CA, USA). PMA and ionomycin were pharmacological T-cell-activating agents that mimicked signals generated by T-cell receptor (TCR) complex and had the advantage of stimulating T cells of any antigen specificity. Monensin was used to block intracellular transport mechanisms, thereby leading to the accumulation of cytokines in the cells. After incubation, the cells were stained with PE-Cy5-conjugated anti-CD4 monoclonal antibodies at room temperature in the dark for 20 min. The cells were then stained with fluorescein isothiocyanate-conjugated anti-interferon (IFN)-γ monoclonal antibodies, phycoerythrin-conjugated anti-IL-17 monoclonal antibodies and allophycocyanin-conjugated anti-IL22 monoclonal antibodies after fixation and permeabilization (eBioscience, San Diego, CA, USA). To enable correct compensation and to confirm antibody specificity, isotype controls were used. Stained cells were analyzed by flow cytometry using a FACScan cytometer equipped with CellQuest software (BD Biosciences, Franklin Lakes, NJ, USA).

### Enzyme-linked immunosorbent assay (ELISA)

Peripheral blood and bone marrow blood were collected into heparin-anticoagulant vacutainer tubes. Plasma was obtained by centrifugation and stored at −80 °C for the determination of cytokines. IL-22, IL-17 and IFN-γ levels were determined with an ELISA kit in accordance with the manufacturer’s recommendations (lower detection limit: IL-22, 5 pg/ml; IL-17, 0.5 pg/ml; IFN-γ, 0.99 pg/ml; eBioscience, San Diego, CA, USA).

### Statistical analysis

All tests were performed by SPSS 17.0 software (IBM, Armonk, NY, USA). Results were expressed as means ± standard deviations. Statistical significance was determined by analysis of variance, and difference between two groups was determined by Newman-Keuls multiple comparison test (q test). For comparison of paired samples, Wilcoxon signed rank test was used. Pearson correlation test was used for correlation analysis. P values less than 0.05 were considered statistically significant.

## Results

### Frequencies of Th22 cells in bone marrow blood of RA patients are higher than those in peripheral blood of RA patients

**Figure 1:**
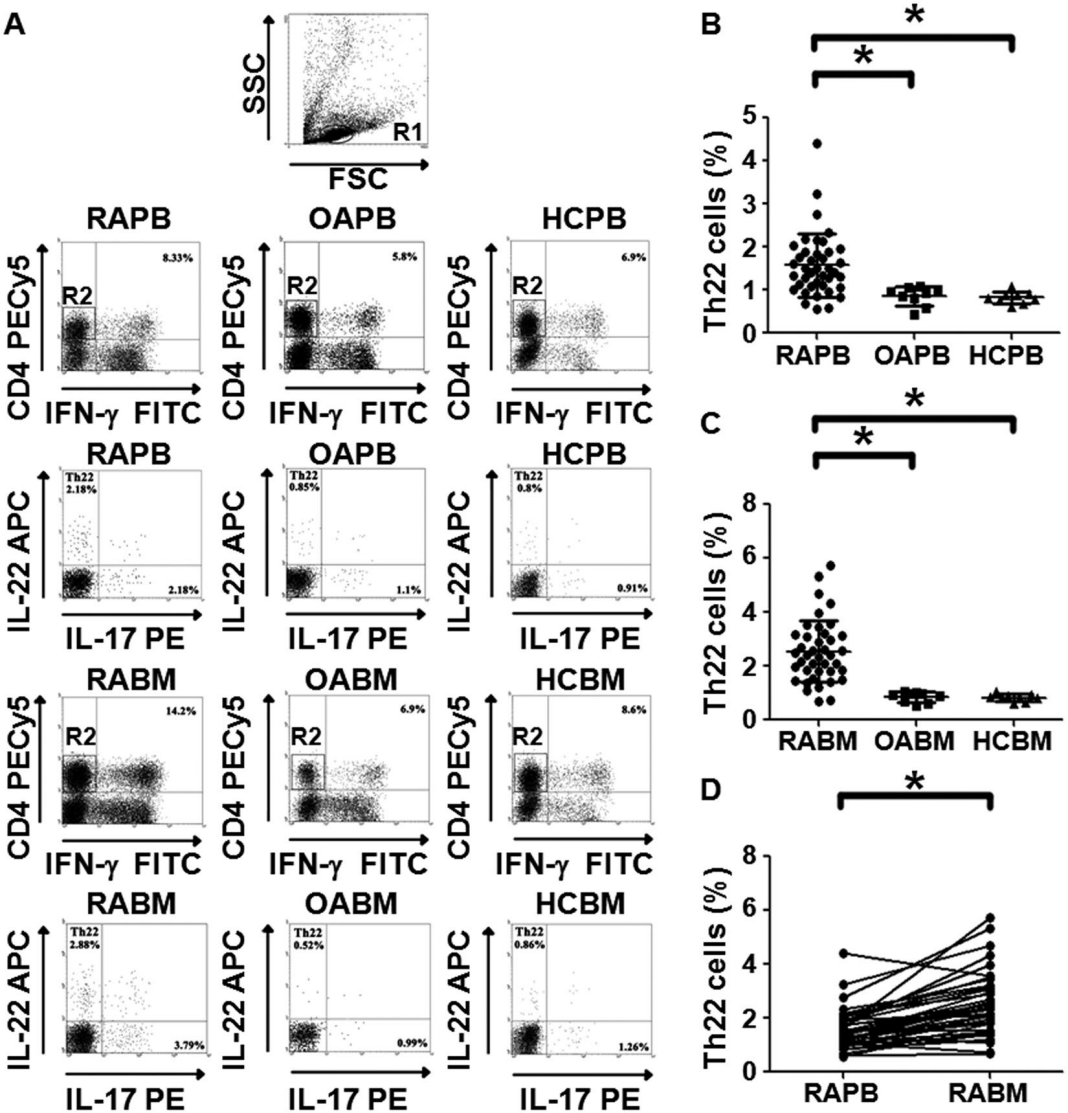
Frequency of Th22 based on cytokine patterns after *in vitro* activation by PMA/ionomycin in short-term cultures. (**A**) Flow cytometry for the detection of the frequency of Th22. (**B**) The percentages of Th22 in peripheral blood from rheumatoid arthritis patients (RAPB), osteoarthritis patients(OAPB) () and healthy controls (HCPB). (**C**) The percentages of Th22 in bone marrow blood from rheumatoid arthritis patients (RABM), osteoarthritis patients(OABM) and healthy controls (HCBM). (**D**) The percentages of Th22 in peripheral blood (RAPB) and bone marrow blood (RABM) from rheumatoid arthritis patients. *P < 0.05.

To analyze the frequency of Th22 in bone marrow blood and peripheral blood after *in vitro* activation by PMA/ionomycin in short-term cultures, flow cytometry was performed. Th22 was defined as CD4+ IFNγ-IL17-IL-22+ T cells to exclude Th1 or Th17 cells. The data showed that the percentage of Th22 cells in peripheral blood from RA patients was significantly elevated compared to that in peripheral blood from OA patients (P = 0.003 < 0.05) or healthy controls (P = 0.002 < 0.05) (Fig. 1A and B), and the percentage of Th22 cells in bone marrow blood from RA patients was significantly higher than that in bone marrow blood from OA patients (P < 0.001) or healthy controls (P < 0.001) (Fig. 1A and C). Furthermore, the percentage of Th22 cells in bone marrow blood from RA patients was significantly higher than that in the paired peripheral blood from RA patients (P < 0.001) (Fig. 1D). These results suggest that frequencies of Th22 cells in bone marrow blood of RA patients are higher than those in peripheral blood of RA patients.

### Percentages of Th17 and Th1 cells in bone marrow blood of RA patients are significantly increased compared with those in peripheral blood of RA patients

**Figure 2:**
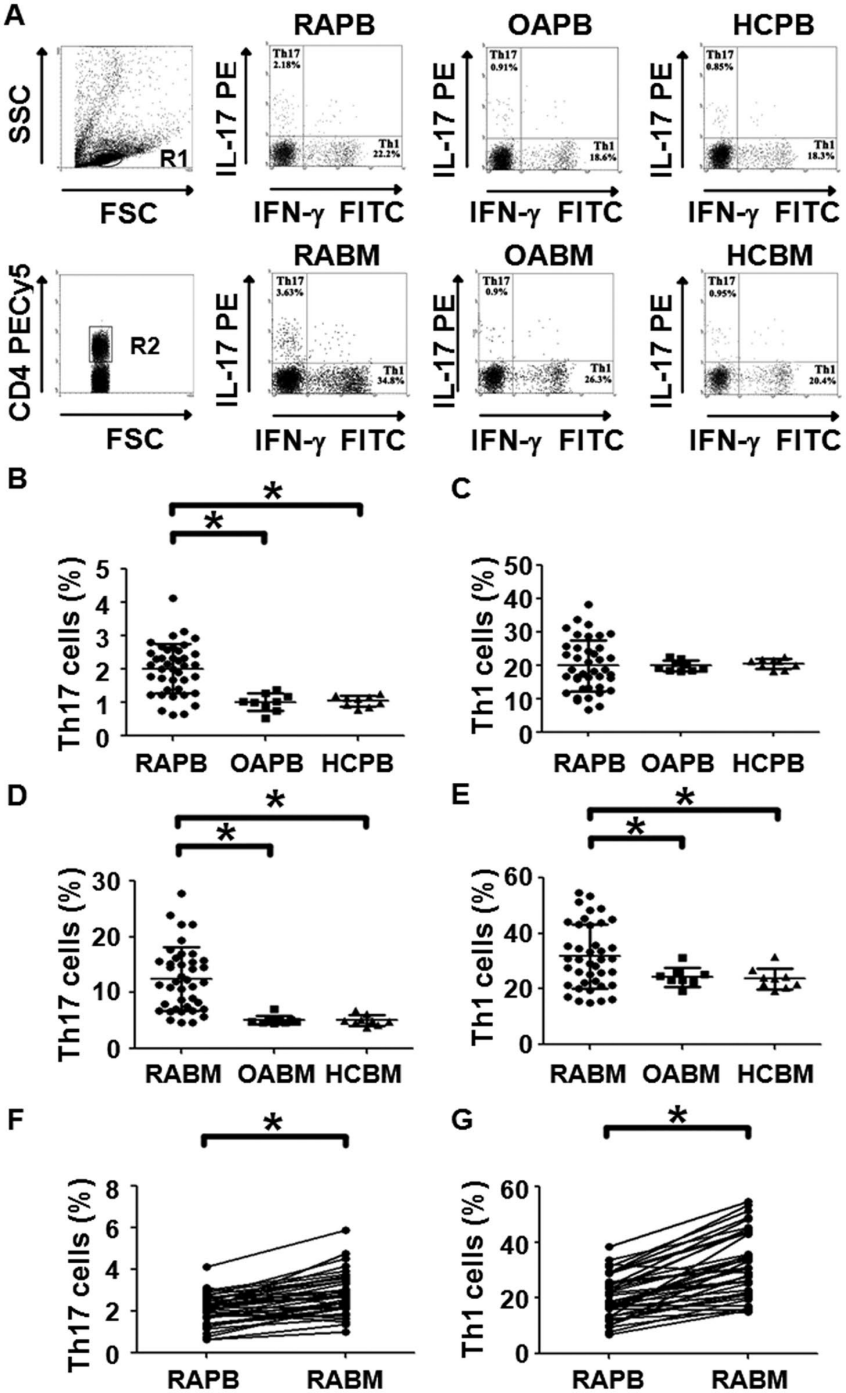
Frequencies of Th17 and Th1. (**A**) Flow cytometry for the detection of the frequencies of Th17 and Th1. (**B**) The percentage of Th17 in peripheral blood from rheumatoid arthritis patients (RAPB), osteoarthritis patients(OAPB) and healthy controls (HCPB). (**C**) The percentage of Th1 in peripheral blood from rheumatoid arthritis patients (RAPB), osteoarthritis patients(OAPB) and healthy controls (HCPB). (**D**) The percentages of Th17 in bone marrow blood from rheumatoid arthritis patients (RABM), osteoarthritis patients(OABM) and healthy controls (HCBM). (**E**) The percentages of Th1 in bone marrow blood from rheumatoid arthritis patients (RABM), osteoarthritis patients(OABM) and healthy controls (HCBM). (**F**) The percentages of Th17 in peripheral blood (RAPB) and bone marrow blood (RABM) from rheumatoid arthritis patients. (**G**) The percentages of Th1 in peripheral blood (RAPB) and bone marrow blood (RABM) from rheumatoid arthritis patients. *P < 0.05.

To detect Th17 and Th1 cells, flow cytometry was used, and Th17 was defined as CD4^+^IFNγ^−^IL17^+^ T cells. The data showed that the percentage of Th17 cells in peripheral blood from RA patients was significantly higher than that in OA patients (P < 0.001) or healthy controls (P < 0.001) (Fig. 2A and B). However, the percentage of Th1 cells in the peripheral blood was not significantly different among all groups (Fig. 2A and C). In addition, the percentage of Th17 cells in bone marrow blood from RA patients was significantly higher than that in bone marrow blood from OA patients (P < 0.001) or healthy controls (P < 0.001) (Fig. 1A and D). Of note, the percentage of Th1 cells in bone marrow blood of RA patients was significantly elevated compared to that of OA patients (P = 0.044 < 0.05) and healthy controls (P = 0.031 < 0.05) (Fig. 2A and E). Furthermore, the percentages of Th17 and Th1 cells in bone marrow blood from RA patients were significantly higher than those in the paired peripheral blood from RA patients, respectively (P < 0.001) (Fig. 2F and G). These results indicate that the percentages of Th17 and Th1 cells in bone marrow blood of RA patients are significantly increased compared with those in peripheral blood of RA patients.

### Plasma levels of IL-22 and IL-17 but not IFN-γ in peripheral blood and bone marrow blood of RA patients are elevated than those of OA patients and healthy controls

**Figure 3:**
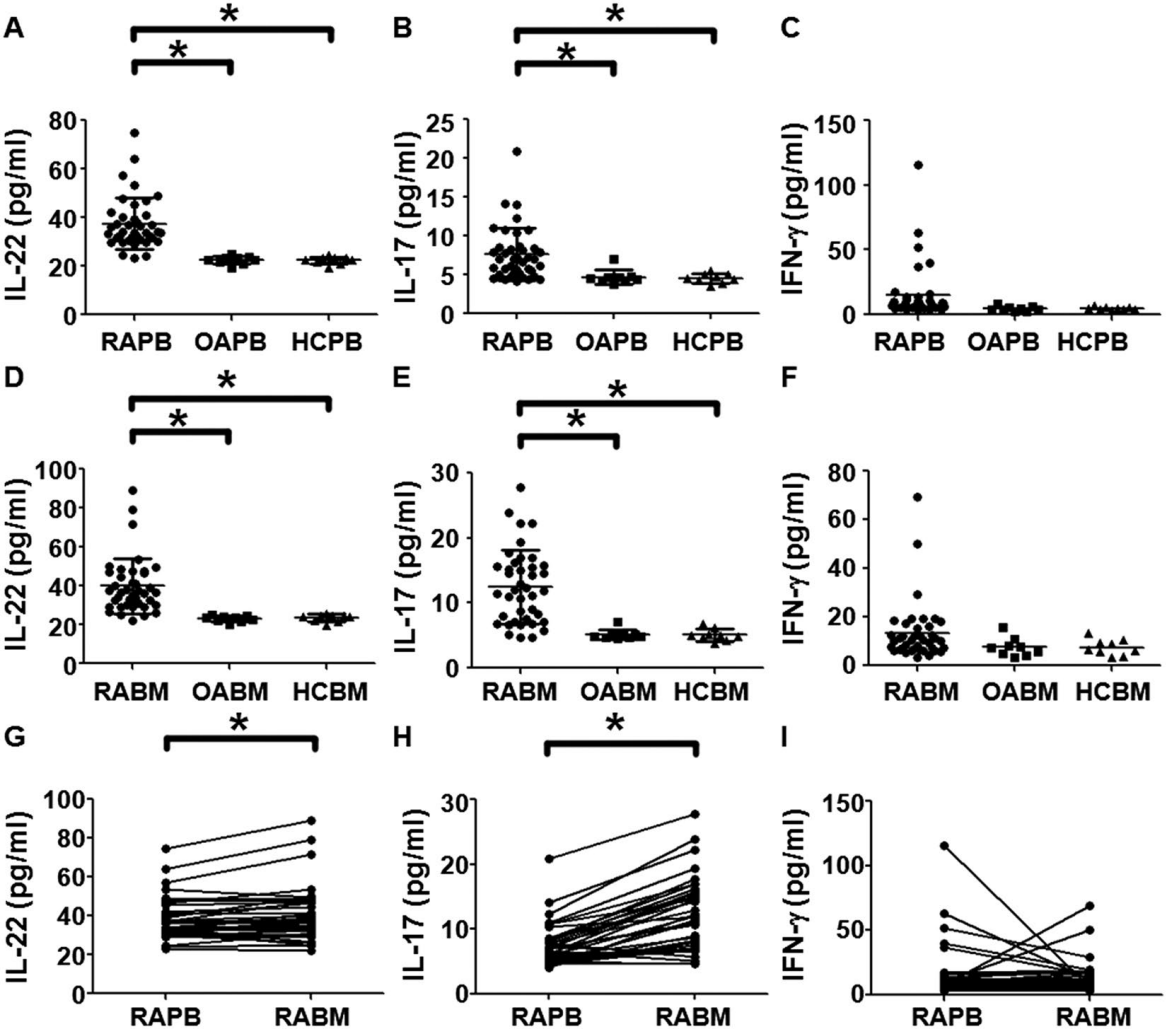
Concentrations of inflammatory cytokines related with T-helper cells. The concentrations of (**A**) IL-22, (**B**) IL-17 and (**C**) IFN-γ in peripheral blood from rheumatoid arthritis patients (RAPB), osteoarthritis patients(OAPB) and healthy controls (HCPB). The concentrations of (**D**) IL-22, (**E**) IL-17 and (**F**) IFN-γ in bone marrow blood from rheumatoid arthritis patients (RAPB), osteoarthritis patients(OAPB) and healthy controls (HCPB). The concentrations of (**G**) IL-22, (**H**) IL-17 and (**I**) IFN-γ in peripheral blood and bone marrow blood from rheumatoid arthritis patients.

To determine the concentrations of IL-22, IL-17 and IFN**-**γ in plasma from both peripheral blood and bone marrow blood, ELISA was carried out. The plasma levels of IL-22 and IL-17 in peripheral blood from RA patients were significantly higher compared with those from OA patients (P < 0.05) and healthy controls (P < 0.05), respectively (Fig. 3A and B). By contrast, the plasma level of IFN-γ in peripheral blood was not significantly different among all groups (Fig. 3C). In addition, the plasma levels of IL-22 and IL-17 in bone marrow blood from RA patients were significantly elevated compared with OA patients (P < 0.001) and healthy controls (P < 0.001) (Fig. 3D and E). However, the plasma level of IFN-γ in bone marrow blood was not significantly different among all groups (Fig. 3F). Of note, the plasma levels of IL22 and IL17 in bone marrow blood from RA patients were significantly higher than those in the paired peripheral blood from RA patients (P < 0.05) (Fig. 3G and H). On the contrary, plasma level of IFN-γ in bone marrow blood from RA patients was insignificantly lower than that in the paired peripheral blood from RA patients (P > 0.05) (Fig. 3I). These results suggest that the plasma levels of IL-22 and IL-17 but not IFN-γ in peripheral blood and bone marrow blood of RA patients are elevated than those of OA patients and healthy controls.

### Th22 and Th17 cells are positively correlated with IL-22 and IL-17 in both peripheral blood and bone marrow blood, respectively, but Th1 cells are only positively correlated with IFN-γ in peripheral blood

**Figure 4:**
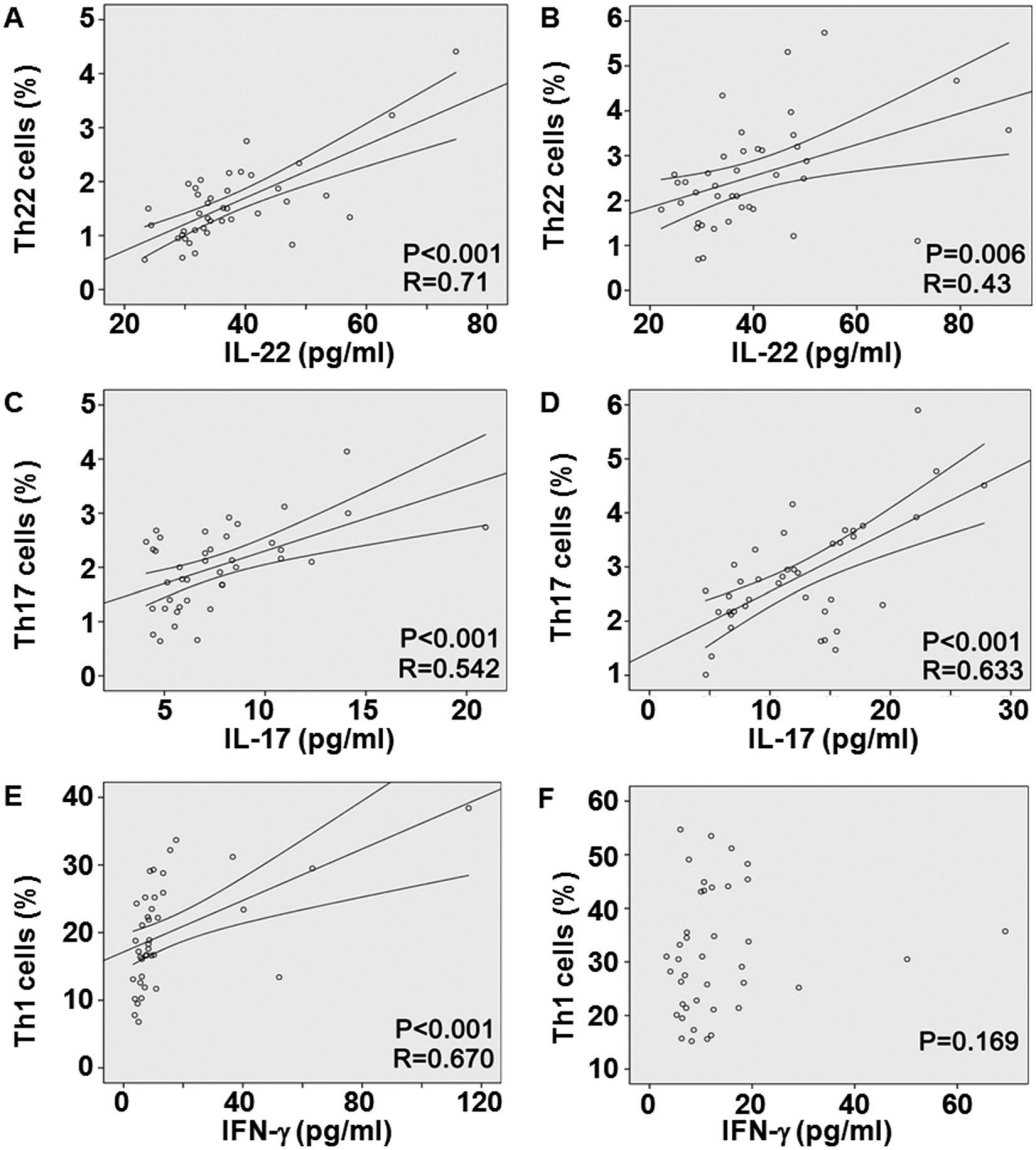
Correlation between the T helper cell subsets and their effective cytokines. Pearson correlation test was employed to study the correlation. (**A**,**B**) Correlation of Th22 with IL-22 in (**A**) peripheral blood and (**B**) bone marrow blood of rheumatoid arthritis patients. (**C**,**D**) Correlation of Th17 with IL-17 in (**C**) peripheral blood and (**D**) bone marrow blood of rheumatoid arthritis patients. (**E**–**F**) Correlation of Th1 with IFN-γ in (**E**) peripheral blood and (**F**) bone marrow blood of rheumatoid arthritis patients.

To identify the relationships between the three T helper cell subsets and their effective cytokines both in peripheral blood and bone marrow blood of RA patients, Pearson correlation test was employed. In RA patients, positive correlations were found between the frequency of Th22 cells and plasma level of IL-22 in both peripheral blood (r = 0.71, P < 0.001) (Fig. 4A) and bone marrow blood (r = 0.43, P = 0.006) (Fig. 4B) of RA patients. Consistently, Th17 cells showed a positive correlation with IL-17 in peripheral blood (r = 0.542, P < 0.001) (Fig. 4C) and bone marrow blood (r = 0.633, P < 0.001) (Fig. 4D) of RA patients. Although there was a positive correlation between Th1 cells and IFN-γ level in peripheral blood (r = 0. 67, P < 0.001) (Fig. 4E), but Th1 cells did not show a statistical correlation with IFN-γ level in bone marrow blood (P = 0.169) (Fig. 4F). These results indicate that Th22 and Th17 cells are positively correlated with IL-22 and IL-17 in both peripheral blood and bone marrow blood, respectively, but Th1 cells are only positively correlated with IFN-γ in peripheral blood.

### Th22, Th17 and Th1 cells in bone marrow blood of RA patients are positively correlated with each other

**Figure 5:**
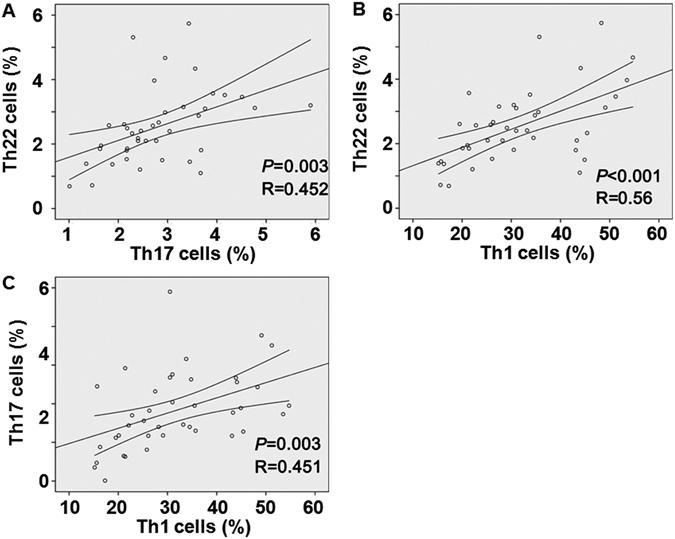
Correlation amongTh22, Th17 and Th1 cells in bone marrow of rheumatoid arthritis patients. Pearson correlation test was employed to study the correlation. (**A**) Correlation of Th22 with Th17. (**B**) Correlation of Th22 with Th1. (**C**) Correlation of Th17 with Th1.

To study the correlation amongTh22, Th17 and Th1 cells in bone marrow blood of RA patients, Pearson correlation test was conducted. The data showed that there were positive correlations between Th22 cells and Th17 cells (r = 0.452, P = 0.003) (Fig. 5A), between Th22 cells and Th1 cells (r = 0.56, P < 0.001) (Fig. 5B), and between Th17 cellsTh1 cells (r = 0.451, P = 0.003) (Fig. 5C). The result suggests that Th22, Th17 and Th1 cells in bone marrow blood of RA patients are positively correlated with each other.

### Th22, Th17 and Th1 cells and plasma levels of IL-22 and IL-17 in bone marrow are positively correlated with DAS28

**Figure 6:**
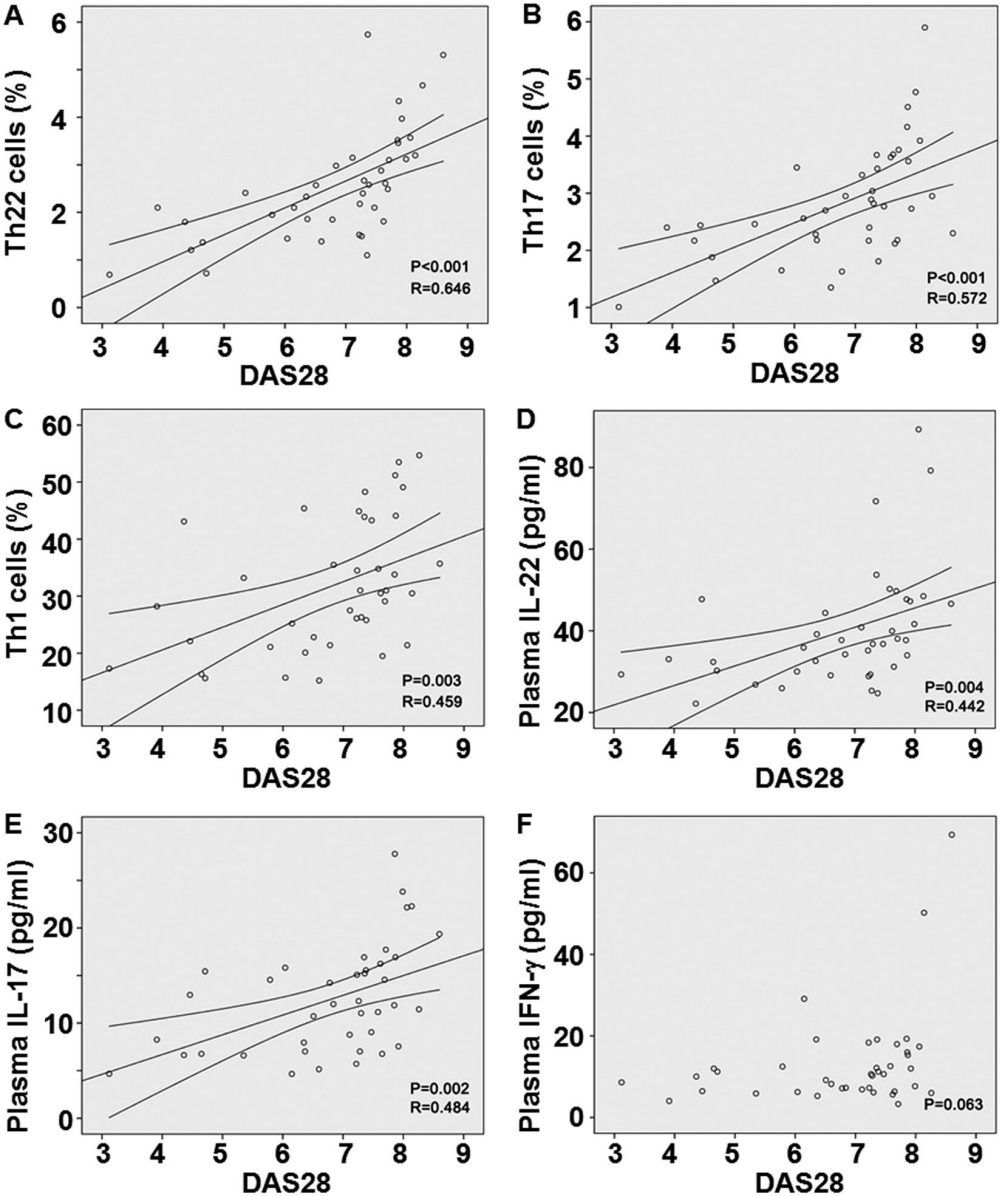
Correlation of T helper cell subsets or their main cytokines in bone marrow with Disease Activity Score in 28 joints. Correlation of (**A**) Th22, (**B**) Th17, (**C**) Th1, (**D**) plasma IL-22 concentration, (**E**) plasma IL-17 concentration, and (**F**) plasma IFN-γ concentration with Disease Activity Score in 28 joints.

To understand how T helper cell subsets or their main cytokines in bone marrow blood are related with DAS28, Pearson correlation test was also used. In patients with RA, there were positive correlations between Th22, Th17 or Th1 cells of bone marrow blood and DAS28 (r = 0.646, P < 0.001; r = 0.572, P < 0.001;and r = 0.459, P = 0.003, respectively) (Fig. 6A–C). Consistently, positive correlations were found between the levels of plasma IL-22 or IL-17 from bone marrow blood and DAS28 (r = 0.442, P = 0.004; and r = 0.484, P = 0.002, respectively) (Fig. 6D and E). However, the plasma level of IFN-γ from bone marrow blood was not significantly correlated with DAS28 (P = 0.063) (Fig. 6F). The result indicates that Th22, Th17 and Th1 cells and plasma levels of IL-22 and IL-17 are positively correlated with DAS28.

## Discussion

CD4+ T cells are central for the development of RA^31^. Th1, Th17 and Th22 cells, the subsets of CD4+ T cells, play primary pathogenic roles in RA^7, 19, 24^. Although these T helper subsets in synovial fluid and peripheral blood of patients with RA have been well studied, little is known about these cells in bone marrow in RA. Bone marrow may have particular role in the pathogenesis of RA.

In the present study, elevated frequencies of Th22 cells are observed in bone marrow blood from RA patients in comparison to OA patients or healthy individuals, indicating the existence of inflammatory environment in subchondral bone region of destructive joints. Consistent with the results of Th22 cells, the level of IL-22 was significantly elevated in plasma from bone marrow blood of RA patients. Furthermore, positive correlations between Th22 and IL-22 suggest that the levels of IL-22 are elevated with the increase of Th22 cells in both peripheral blood and bone marrow blood from RA patients. The pro-inflammatory effects of Th22 cells are synergistically dependent on IL-22 and TNF-α^32^. As the main effective cytokine of Th22 cells, IL-22 promotes inflammatory responses in RA synovial tissues by inducing the expression of inflammatory chemokines^33^. Furthermore, Lies *et al*. report that IL-22 promotes osteoclastogenesis in arthritis of mice induced by collagen^14^. Although Th17 cells express IL-22, the percentage of Th17 cells that express IL-22 is lower compared to Th22 cells. TNF-α, another important pro-inflammatory cytokine of Th22 cells, contributes to inflammatory disorders and osteoclastogenesis through several mechanisms in RA. More importantly, the frequency of Th22 cells and the level of IL-22 in bone marrow blood were significantly higher than that in peripheral blood of RA patients. Meaningfully, the percentage of Th22 cells in bone marrow was higher than that in the paired peripheral blood in most RA patients (32 out of 40). Th22 cells are reported to co-express chemokine receptor CCR6^12^. Correspondingly, up-regulated expression of CCL20^34^, the ligand of CCR6, is discovered in subchondral bone tissue biopsies of RA patients^35^, facilitating the migration of Th22 cells to bone region. Cytokines like IL-6 and TNF-α, which are necessary for Th22 differentiation^12^, are elevated in bone marrow from RA patients^36, 37^. Therefore, the increased proliferation *in situ* and the migration from peripheral blood may attribute to abnormal accumulation of Th22 cells in bone marrow. The elevation of Th22 cells in bone marrow from RA patients may accelerate inflammatory process and lead to inflammatory bone destruction *in situ*. OA patients also suffer from joints destruction, but Th22 cells and related cytokines in bone marrow of OA patients were similar to that of healthy controls, suggesting that different pathogenic mechanism may be involved in joint destruction between RA and OA. The homing of Th22 cells in bone marrow of RA patients may lead to local inflammatory bone destruction by promoting the production of osteoclastogenic cytokines.

Th17 cells, another important pro-inflammatory T helper subset, were also significantly higher in bone marrow from RA patients compared with OA patients and healthy individuals. In line with results of Th22 cells, Th17 cells were markedly elevated in bone marrow in comparison to paired peripheral blood of most RA patients (36 out of 40). Th17 cells also co-express chemokine receptor CCR6^38^, which contributes the homing of Th17 cells to bone marrow of RA patients. IL-17 is a critical effective cytokine of Th17 cell subset. With the elevation of Th17 cells in both peripheral blood and bone marrow, the levels of IL-17 are also increased in RA patients. Our results showed a higher level of IL-17 in bone marrow plasma than in peripheral blood of RA patients, suggesting that excessive IL-17 is produced locally in bone marrow of inflamed RA joints. IL-17 derived from T cells of bone marrow contributes to joint degradation, including cartilage and bone destruction^39^. In the presence of other cytokines, IL-17 promotes bone destruction much more seriously^40^. IL-17 can exert its pro-inflammatory function more effectively *in situ* by synergizing with the elevated IL-22 in bone marrow. As a result, the accumulation of Th17 cells in subchondral bone marrow may lead to more severe bone damage.

Consistent with our previous results^25^, Th1 cells in peripheral blood showed no significant difference among RA, OA and healthy individuals in the present study. However, frequency of Th1 cells in bone marrow from RA patients was significantly increased compared with that from OA patients. Consistent with the results of Th22 and Th17 cells, Th1 cells were also markedly elevated in bone marrow compared to paired peripheral blood from most RA patients (38 out of 40). To further understand the effective cytokine of Th1 cells in bone marrow, we examined the plasma concentrations of IFN-γ in peripheral blood and bone marrow. No significant difference of plasma IFN-γ level in both peripheral blood and bone marrow blood was observed among the three groups in our study. Furthermore, there was no statistical difference in plasma IFN-γ level between peripheral blood and bone marrow blood in RA patients, suggesting that IFN-γ cannot effectively play its anti-osteoclastogenic role in bone marrow of RA patients. RA is primarily driven by Th1 cells, and the activated Th1 cells lead to bone destruction regulated by osteoclasts. IFN-γ, produced mainly by Th1 cells, is an anti-osteoclastogenic cytokine. However, Th1 cells may enhance osteoclastogenesis through many other ways in RA. For example, Th1 cells can co-express several pro-inflammatory osteoclastogenic cytokines, such as TNF-α, IL-17 and IL-22. In addition, the increasing Th1 cells in bone marrow of RA express more RANKL that can induce the formation of osteoclast and activate mature osteoclasts. Th1 cells also predominate in the joints of RA patients^24^. The elevation of Th1 cells in the adjacent compartment of joints, including both synovial fluids and bone marrow, suggests the bidirectional erosion of bone and cartilage in rheumatoid joints.

Bugatti *et al*. report that lymphoid accumulates in subchondral area of joints in RA^41^. Additionally, increased numbers of CD4 T cells in bone marrow of patients with RA have also been reported^42^. In the present study, our analysis demonstrates the aggregation of Th22, Th17 and Th1 cells in bone marrow of RA patients. Moreover, there were positive correlations among Th22, Th17 and Th1 cells in bone marrow of RA patients, suggesting that the three T helper cells are all increased in bone marrow. As the main inflammatory T helper subsets, Th17 and Th22 cells are usually increased in areas with persistent inflammation, and our observation strongly supports the idea that more severe inflammatory process occurs in bone marrow from destructive joints of RA patients. Activated T-helper cells are important sources of osteoclastogenic cytokines in RA^43^. According to the results of MRI, periarticular bone marrow is affected early in the disease course, and leads to local bone loss in patients with early RA^27, 28^. Moreover, Katsuyuki *et al*. find that pathological damage exists in subchondral bone of RA patients before obvious inflammation of synovial membrane^44^. Serena *et al*. report that lymphoid accumulation is related to increased osteoclast recruitment in subchondral bone^41^. Consistent with these observations, accumulation of Th22, Th17 and Th1 cells in bone marrow may contribute to bone destruction through the aggregation of osteoclasts *in situ*. All the results suggest that inflammation in bone marrow of RA patients occurs and develops independently to some extent. In addition, Th22, Th17 and Th1 cells in bone marrow are also associated with DAS28. This means that higher numbers of Th cells correspond to more joints that suffer from tender and swollen. Furthermore, the extent of focal bone destruction is associated with the severity of RA^45^. The cartilage and bone destruction become more severe and faster during the period of disease activity. Therefore, the profiles of pro-inflammatory T helper cells in bone marrow may reflect the degree of focal bone erosion, as well as the severity and disease activity of RA.

In conclusion, the present study provides detailed profiles of the main pathogenic Th cell subsets and the levels of their effective cytokines in subchondral bone marrow of RA patients. Our study evidences that abnormal T helper cells exist not only in synovial fluid as well as peripheral blood, but also in the subchondral bone marrow of RA patients. In the future, studies on how and when these T helper cells subsets recruit in the subchondral bone marrow may provide more comprehensive understanding of the inflammatory joint destruction in RA. Finally, pathogenic T helper cells in bone marrow may be used as a potential target for immunotherapeutic strategies against inflammatory joint destruction in RA.
